# Preventive Management of a Primary Tooth with Ankylosis

**DOI:** 10.3390/pediatric18020046

**Published:** 2026-03-30

**Authors:** Yumeng Wu, Yandi Chen, Qiong Zhang, Yiran Peng, Jing Zou

**Affiliations:** 1 State Key Laboratory of Oral Diseases & National Center for Stomatology & National Clinical Research Center for Oral Diseases, Department of Pediatric Dentistry, West China Hospital of Stomatology, Sichuan University, Chengdu 610041, China; 2 State Key Laboratory of Oral Diseases & National Center for Stomatology & National Clinical Research Center for Oral Diseases & Department of Pediatric Dentistry & Jinjiang Outpatient Department, West China Hospital of Stomatology, Sichuan University, Chengdu 610041, China

**Keywords:** primary molar ankylosis, pre-eruptive intracoronal resorption, interceptive orthodontic treatment, impact of primary molar, preventive dentistry, 3D-printed space maintainer

## Abstract

**Objectives:** This study aimed to investigate preventive management strategies and optimal intervention timing for dental ankylosis of primary teeth complicated by suspected pre-eruptive intracoronal resorption (PEIR), providing an evidence-based framework for clinical diagnosis and management. **Methods**: This case retrospectively reports a 7-year-old patient with an ankylosed mandibular left second primary molar (tooth 75), exhibiting radiographic features suggestive of pre-eruptive intracoronal resorption. The patient was in the mixed dentition stage with dental crowding. Preventive and interceptive orthodontic management was implemented to address space deficiency and guide occlusal development. The timing of extraction and space maintenance of tooth 75 was guided by space regaining, PEIR lesion progression, and crown development of tooth 35. **Results**: The permanent successor of tooth 75 (tooth 35) erupted successfully, dental crowding was alleviated, and a favorable occlusion was established. **Conclusions**: Early diagnosis and timely, individualized intervention for ankylosed primary teeth play an important role in preventing malocclusion and promoting normal eruption of the permanent successor tooth.

## 1. Introduction

Dental ankylosis refers to a direct fusion between the cementum and alveolar bone, resulting in the loss of the periodontal ligament at the affected site [1]. Primary tooth ankylosis is relatively uncommon in clinical practice, with a reported prevalence ranging from 1.3% to 8.9% [2]. Ankylosis most commonly affects mandibular posterior primary teeth [3,4]. Clinically, ankylosed primary teeth typically present with infraocclusion, characterized by an occlusal surface positioned below the normal occlusal plane of adjacent teeth [5]. Depending on the severity, infraocclusion can be classified into mild, moderate, or severe, based on the relationship of the occlusal surface to the adjacent teeth and gingival margin [6]. Ankylosed primary teeth may interfere with the eruption pathway of permanent successor, leading to impaction or ectopic eruption [5,7]. Adjacent teeth may tip into the infraoccluded space, causing loss of arch length, dental asymmetry, and midline deviation [1,6]. Severe infraocclusion may also lead to insufficient alveolar bone height [8]. Primary tooth ankylosis may compromise development of the permanent successors, with possible effects on root formation and crown morphology [3,9]. Therefore, early diagnosis and timely intervention are important to minimize these potential complications [7].

In addition to ankylosis, pre-eruptive intracoronal resorption (PEIR) is another rare dental anomaly. PEIR is a defect of the coronal dentin in unerupted or partially erupted teeth, radiographically presenting as a radiolucency within the coronal dentin adjacent to the enamel–dentin junction [10]. While occasionally seen in permanent teeth, it is extremely rare in the primary dentition [11]. The etiology of PEIR is considered to be associated with abnormal eruption positions and localized pressure, which can disrupt the protective reduced enamel epithelium and initiate a resorptive process [12]. Because PEIR is typically hidden and asymptomatic, it is easily overlooked until eruption, when it progresses to extensive tooth destruction or secondary pulpal infection [13].

This case report presents the preventive and individualized management of an ankylosed mandibular second primary molar (tooth 75), with radiographic features suggestive of pre-eruptive intracoronal resorption (PEIR) in a 7-year-old patient. This report aims to show how early, staged management can preserve the eruption potential of the permanent successor and reduce the risk of future complications.

This case is distinguished by the coexistence of severe ankylosis and a suspected PEIR lesion, individualized extraction timing based on the Nolla stage of the permanent successor, and use of a digital workflow to fabricate a 3D-printed metal space maintainer. This report highlights a preventive and individualized approach to the management of a complex case.

## 2. Materials and Methods

Throughout this report, teeth are identified using the Fédération Dentaire Internationale (FDI) two-digit tooth numbering system.

### 2.1. General Information

A 7-year-old female patient presented with the chief complaint of irregular and crowded dentition following tooth replacement. The patient reported no discomfort and no history of dental trauma or previous orthodontic treatment. The patient was in good general health, with no history of systemic disease or drug allergies. Both parents reported normal dental development, with no family history of similar dental abnormalities. Informed about the proposed treatment at the outset, the parents granted their consent for the procedure.

### 2.2. Diagnostic Records and Clinical Examination

#### 2.2.1. Extraoral Examination

**Figure 1:**
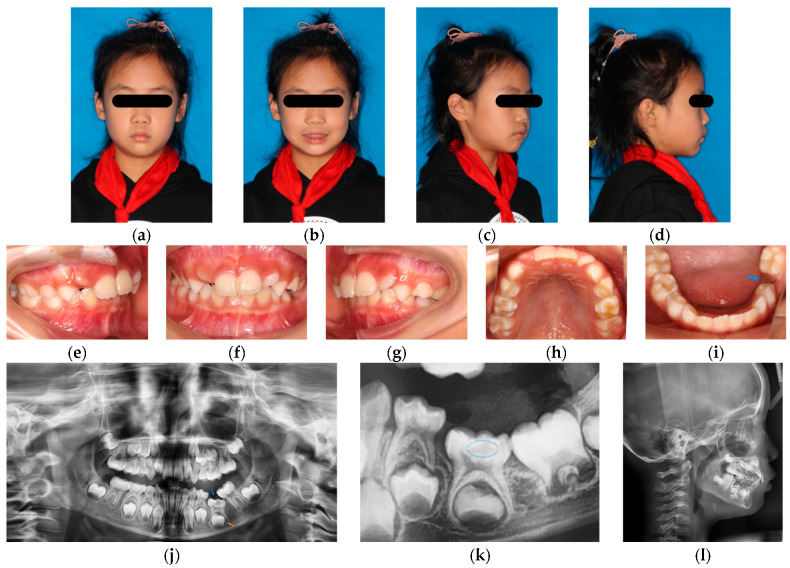
Initial facial photographs, intraoral photographs, and radiographic records of the patient. (**a**–**d**) Initial facial photographs. (**e**–**i**) Initial intraoral photographs showing mixed dentition with dental crowding and tilting of several teeth; the site of tooth 75 is indicated by the blue arrow in figure (**i**). (**j**) Initial panoramic radiograph showing severe infraocclusion of tooth 75 (blue arrow) with an indistinct periodontal ligament space at the mesial root and an intracoronal radiolucency extending into the middle layer of dentin beneath the enamel–dentin junction, and a close relationship between the root bifurcation of tooth 75 and the cortical bone of the 35 tooth bud (orange arrow); tooth 36 exhibits mesial inclination. (**k**) Radiographs obtained two years ago showing a radiolucency beneath the enamel–dentin junction of tooth 75 (blue dashed oval). (**l**) Initial lateral cephalometric radiograph.

The patient exhibited normal growth and development. Facial examination revealed mild facial asymmetry with the chin deviated to the left, and no obvious skeletal or soft tissue abnormalities (Figure 1a–d).

#### 2.2.2. Intraoral Examination

The patient was in the early mixed dentition stage, with tooth 75 not visible intraorally. Tooth 36 exhibited mesial ectopic eruption and tooth 74 exhibited distal tipping, resulting in an approximately 5 mm space between teeth 74 and 36. Tooth 22 presented with mesial rotation, and premature contacts were observed between teeth 22 and 32. Mild crowding was observed in the maxillary dentition (approximately 3 mm), and moderate crowding was present in the mandibular dentition (approximately 6 mm). The mandibular dental midline was deviated approximately 2 mm to the left (Figure 1e–i).

#### 2.2.3. Radiographic Examination

Panoramic radiography revealed severe infraocclusion of tooth 75, with its occlusal surface positioned approximately at the cervical level of adjacent teeth 74 and 36 (Figure 1j). The periodontal ligament space of tooth 75’s mesial root was indistinct, and an intracoronal radiolucency was observed extending to the middle layer of the dentin beneath the enamel–dentin junction. Tooth 36 exhibited mesial and lingual inclination.

A panoramic radiograph taken two years ago provided by the patient’s parents (Figure 1k) showed a less pronounced radiolucency within the crown of tooth 75.

Lateral cephalometric analysis (Figure 1l) demonstrated the following measurements: SNA 80.55°, SNB 75.35°, ANB 5.2°, FMIA 57.8°, FMA 22.16° and IMPA 100.04°. Detailed cephalometric values are presented in Table 1. The cephalometric analysis indicated that the patient was skeletal Class I with a horizontal growth pattern.

According to the clinical findings and radiographic evidence above, the patient was diagnosed as experiencing ankylosis of tooth 75 with severe infraocclusion, suspected pre-eruptive intracoronal resorption, ectopic eruption of tooth 36, mild maxillary crowding and moderate mandibular crowding, and a skeletal Class I pattern with a horizontal growth pattern.

The lesion was identified beneath an apparently intact enamel surface before oral exposure. The tooth had not erupted, making occult caries unlikely. Internal resorption was less likely because the lesion was confined coronally beneath the enamel–dentin junction on panoramic assessment.

### 2.3. Treatment Objectives and Plan

Interceptive orthodontic treatment was initiated during the mixed dentition period. The primary treatment objectives were to correct ectopic eruption of the mandibular first permanent molar, regain and maintain adequate space for the permanent successor tooth (tooth 35), harmonize the arch form, and guide favorable occlusal development.

The treatment plan included maxillary and mandibular arch expansion to address arch form discrepancy and anterior tooth rotation. A localized mandibular fixed appliance was applied to upright tooth 36 and regain the space of tooth 75. Following uprighting of tooth 36, 3D-printed space maintenance was planned to preserve the eruption space for tooth 35.

The progression of the intracoronal lesion of tooth 75 and the developmental stage of tooth 35 were closely monitored. Extraction of tooth 75 was planned at an appropriate stage based on restoration of sufficient space, progression of the intracoronal lesion, and development of tooth 35.

## 3. Results

### 3.1. Treatment Procedures

**Figure 2:**
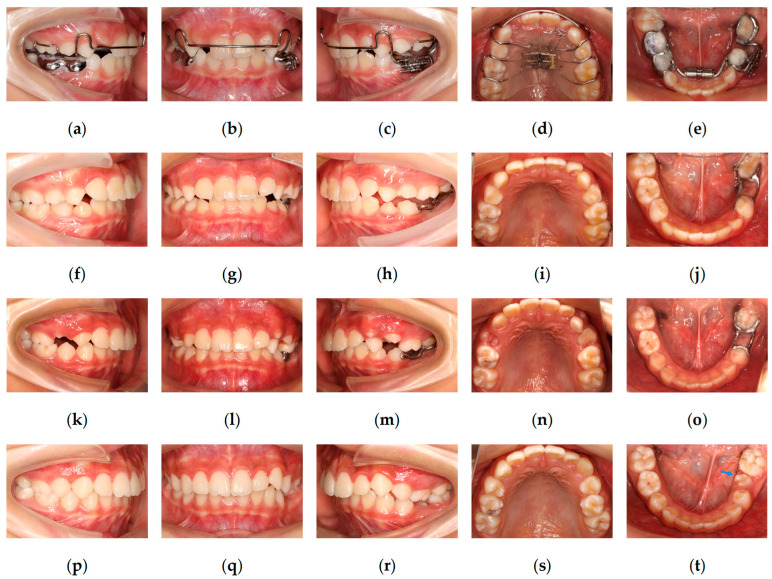
Intraoral photographs during the treatment. (**a**–**e**) Maxillary active expander and mandibular fixed appliance. (**f**–**j**) Intraoral photograph obtained after 15 months of treatment, demonstrating the 3D-printed metal space maintainer for tooth 75 and inter-arch elastics between teeth 26 and 36. (**k**–**o**) Intraoral photographs obtained after 22 months of treatment. (**p**–**t**) Intraoral photograph after 33 months of treatment, demonstrating the eruption of tooth 35 with mild mesial rotation; tooth 35 is indicated by the blue arrow in figure (**t**).

**Figure 3:**
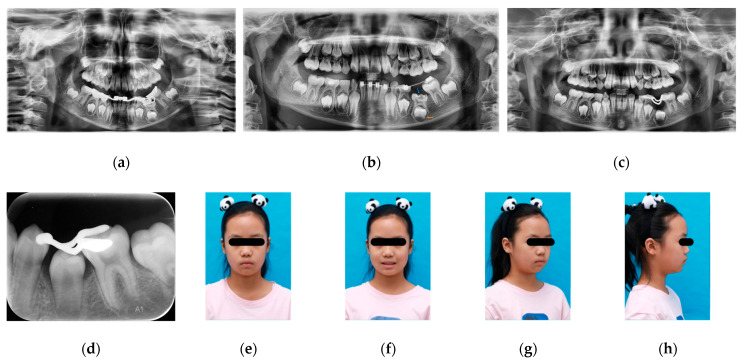
Panoramic radiograph, periapical radiograph and facial photograph during the treatment. (**a**) Panoramic radiograph obtained at treatment initiation. (**b**) Panoramic radiograph obtained after 8 months of treatment, showing a radiolucency within the crown of tooth 75 extending to the deep layer of dentin (blue arrow), and the root of tooth 35 reaching Nolla stage VI (orange arrow). (**c**) Panoramic radiograph obtained after 12 months of treatment, demonstrating uprighting of teeth 74 and 36, with no bony coverage above tooth 35. (**d**) Periapical radiograph obtained after 31 months of treatment, demonstrating the root development of tooth 35 at Nolla stage VII–VIII. (**e**–**h**) Facial photographs obtained after 33 months of treatment.

Maxillary expansion was initiated using a removable appliance, with a double helical lingual spring positioned on the lingual side of tooth 22 to facilitate anterior alignment. A mandibular fixed appliance was used for expansion to correct the discrepancy between the maxillary and mandibular arch forms. A screw-type expansion appliance was applied to upright tooth 36 and apply distalizing force (Figure 2a–e and Figure 3a).

After four months of treatment, arch expansion was largely completed. Due to difficulties in appliance adjustment and patient tolerance, the mandibular expansion appliance was removed. Maxillary expansion was discontinued. A localized fixed appliance was subsequently bonded to align the mandibular anterior teeth. A helical spring was applied to further upright tooth 36 and enhance distalization.

After eight months of treatment, the alleviation of tooth 36 mesial inclination was observed, and tooth 74 was uprighted, resulting in restoration of the eruption space of tooth 75. Radiographic examination demonstrated progression of the intracoronal radiolucent lesion of tooth 75, extending into the deep dentin layer. Concurrently, root development of tooth 35 had progressed to Nolla stage VI, indicating completion of crown calcification (Figure 3b). Based on the regaining of adequate space, progression of the intracoronal lesion, and completion of crown development of the permanent successor, extraction of tooth 75 was performed after removing the mandibular fixed appliance. After satisfactory healing of the extraction site, a 3D-printed metal space maintainer was fabricated to preserve the space of tooth 75. Buttons were bonded to the buccal surface of tooth 26 and the lingual surface of tooth 36, and cross-elastics (1/8-inch, 3.5 oz) were applied to correct lingual inclination of tooth 36 (Figure 2f–j and Figure 3c).

At 22 months after treatment initiation, a favorable occlusion was achieved between teeth 26 and 36. Tooth 74 exhibited grade III mobility and was approaching exfoliation. Accordingly, the space maintainer of tooth 75 was removed, tooth 74 was extracted, and buttons on teeth 36 and 26 were removed. Following eruption of tooth 34, a new 3D-printed metal space maintainer was fabricated, and eruption of the successor (tooth 35) continued to be monitored (Figure 2k–o).

At 31 months after treatment initiation, periapical radiographs demonstrated that root development of tooth 35 had progressed to Nolla stages VII–VIII (Figure 3d), indicating advanced root formation. The space maintainer was removed.

At 33 months after treatment initiation, tooth 35 had erupted into the oral cavity, presenting with mild mesial rotation. The patient was placed under continued observation with regular follow-up examinations (Figure 2p–t and Figure 3e–h).

### 3.2. Treatment Outcomes, Follow-Up, and Prognosis

The patient initially presented with malocclusion associated with ankylosis of tooth 75. Following interceptive management, tooth 36 was successfully uprighted, the ankylosed primary molar tooth 75 was extracted, and the adequate eruption space for the successor permanent tooth was restored. Tooth 35 subsequently erupted. The arch form was harmonized, dental crowding was alleviated, and a favorable occlusion was established.

**Figure 4:**
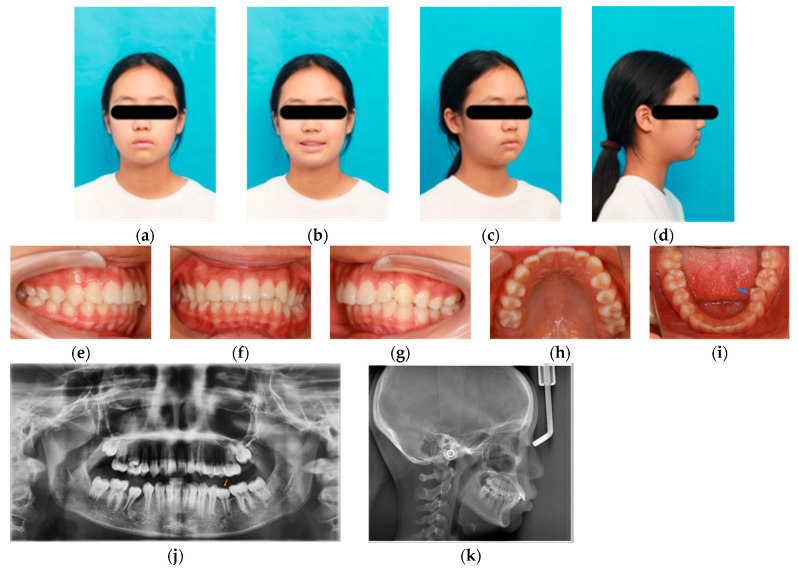
Facial, panoramic, and intraoral photographs obtained at the 2-year follow-up after treatment. (**a**–**d**) Facial photographs at the 2-year follow-up. (**e**–**i**) Intraoral photographs at the 2-year follow-up, showing eruption of tooth 35 to the occlusal plane with mesial rotation and slight lingual inclination; tooth 35 is indicated by the blue arrow in figure (**i**). (**j**) Panoramic radiograph at the 2-year follow-up; tooth 35 is indicated by the orange arrow. (**k**) Lateral cephalometric radiograph at the 2-year follow-up.

At the 2-year follow-up after completion of treatment (Figure 4), tooth 35 had erupted to the occlusal plane, presenting with mild mesial rotation and slight lingual inclination. Cephalometric changes relative to pretreatment are presented in Table 1. These measurements demonstrated a slight reduction in the ANB angle (from 5.2° to 4.52°), shifting closer to the mean average. The slight change in ANB angle is probably attributed to the maxillofacial growth of the patient. The patient retained a Class I skeletal pattern and horizontal growth pattern throughout the interceptive treatment and the follow-up. The occlusion remained stable, and the patient and her parents were satisfied with the current outcomes.

## 4. Discussion

Ankylosed primary teeth may present as a delayed eruption or absence from the dental arch, and may lead to occlusal disturbances, insufficient alveolar bone development, space loss, and tipping of adjacent teeth [3,6], while also affecting distal teeth [1,14]. Radiographic evaluation is required to confirm the diagnosis [15]. Quantitative evidence has confirmed the relationship between the degree of infraocclusion and adjacent tooth inclination, as well as alveolar bone height alterations [8].

The etiology of dental ankylosis in primary teeth remains unclear. Periodontal ligament trauma, local infection, or localized irritation have been proposed as potential contributing factors [2]. Inflammatory responses related to disruption of dental follicle remnants and epithelial rests of Malassez have been suggested [16,17]. A familial tendency has been reported [18], although no parental history of eruption abnormalities was identified in the present case.

PEIR occurs in unerupted teeth without bacterial exposure and represents a resorptive process rather than an infectious one. Once the affected tooth erupts or becomes exposed to the oral environment, the lesion is highly susceptible to rapid secondary caries and pulpal infection [19]. When PEIR lesions are confined to the middle dentin layer or less, careful monitoring with planned intervention may be appropriate; however, once the lesion extends deeper, immediate treatment is recommended to prevent pulpal involvement [11]. If progression results in pulpal necrosis and periapical infection, clinical complications such as pain and acute inflammation may develop, and the associated inflammatory process may potentially compromise the development and eruption pathway of the permanent tooth.

PEIR occurs predominantly in posterior permanent teeth and is rare in primary dentition [11]. However, Seow et al. suggested that many PEIR lesions in primary teeth may be underdiagnosed because panoramic radiographs are not routinely obtained during the eruption period in young children. Consequently, such lesions may only be detected after eruption, when they present as cavitated caries or pulpal infection [13]. The etiology of PEIR appears to be multifactorial. Abnormal eruption position is considered the most significant contributing factor, as localized pressure may disrupt the integrity of the reduced enamel epithelium, thereby enabling osteoclast-like cells to access the dentin and initiate resorption [12].

In the present case, the pathogenic relationship between severe ankylosis and the progressing PEIR lesion warrants critical discussion. Mechanically, the severe infraocclusion of tooth 75 likely generated abnormal localized pressure and contributed to the progression of the intracoronal resorption lesion. Furthermore, a biological link between these two anomalies is highly probable. As previously noted, the ankylosis process is often accompanied by localized inflammatory responses [16,17]; it is biologically conceivable that these inflammatory mediators altered the local microenvironment, promoting the resorptive process within the crown. Enamel developmental defects, delayed tooth development, and systemic conditions have also been proposed as potential contributing factors, although the underlying mechanisms remain unclear [20,21].

Management of ankylosed primary teeth is largely determined by the severity of infraocclusion and associated conditions, with the timing of intervention being critical. Some infraoccluded primary teeth have been reported to be diagnosed as late as 13.6 years of age [3,22]. At this stage, the roots of the permanent successors are usually relatively mature, and orthodontic traction is often required after extraction of the impacted primary teeth [23]. Early extraction is recommended for severe infraoccluded ankylosed second primary molars, particularly when accompanied by hard tissue or pulpal diseases, to prevent vertical alveolar bone loss or occlusal abnormalities [20,21], as in the present case with PEIR.

Given this complex dual pathology, conservative observation was dismissed because the severe infraocclusion had already resulted in significant space loss, necessitating orthodontic uprighting of adjacent teeth [24]. Other potential alternatives reported in the literature include restorative treatment of PEIR lesions [25], surgical luxation for infraoccluded ankylosed primary molars [26], and root-retaining conservative procedures such as decoronation [27]. Restorative treatment of the PEIR lesion was not feasible because the submerged tooth prevented adequate moisture control. Additionally, PEIR exhibits a self-limiting nature; moderate and non-progressing lesions typically do not require intervention [11]. Surgical luxation was excluded due to risk of damaging the permanent bud, and the evidence supporting its use in primary molars remains limited [26]. Decoronation was mainly applied in permanent tooth ankylosis [27]. In this case, preservation of alveolar bone through root retention was unnecessary because the permanent successor was present. Above all, the primary goal was to ensure normal eruption of the successor tooth.

In the present case, although the dual pathology strongly indicated extraction, several clinical limitations precluded immediate surgical intervention. At the initial stage, the space of tooth 75 was limited and surgical access was restricted. In addition, the close anatomical relationship between the ankylosed primary molar and the permanent successor posed a risk of damage, particularly when the crown calcification was incomplete [9,28]. Therefore, we first uprighted the adjacent teeth and regained the space, while closely monitoring the development of the permanent successor and the intracoronal lesion of tooth 75. After 8 months of treatment, tooth 35 had reached Nolla stage VI, with complete crown calcification, making extraction of tooth 75 relatively safe. Meanwhile, progression of the PEIR lesion in tooth 75, with radiolucency extending into the deep layer of dentin, further supported extraction at this stage to prevent subsequent pulpal infection. Importantly, timely removal of tooth 75 before infection develops may help avoid potential adverse effects on tooth 35, given their close anatomical relationship in this case.

After extraction of severely infraoccluded ankylosed primary molars, appropriate space management is required [7,29,30]. In some cases, eruption of the successor permanent teeth occurs when adequate space is maintained [31,32]. However, if severe infraocclusion has significantly altered the eruption path of the permanent successor, orthodontic traction may be required to guide eruption [26,33]. Some studies have also reported that creating sufficient eruption space for impacted infraoccluded primary molars may allow spontaneous eruption [34,35]. Treatment strategies for children with infraoccluded primary molars should be individualized based on the severity of infraocclusion and the patient’s clinical characteristics.

In this case, we preserved the space of tooth 75 using a 3D-printed metal space maintainer. Considering the root development of the successor permanent (tooth 35) had progressed only to Nolla stage VI, tooth 35 still had the potential for spontaneous eruption, and long-term space maintenance was anticipated. Therefore, a digital workflow was adopted to fabricate a 3D-printed metal space maintainer. Compared with conventional hand-bent band-and-loop appliances, this approach uses intraoral scanning to obtain digital impressions, effectively avoiding the gag reflex and nausea commonly associated with traditional alginate impressions, thereby improving treatment tolerance and cooperation in young children [36]. Three-dimensional printing devices based on selective laser sintering (SLS) technology have demonstrated excellent clinical performance [37]. Compared with manually fabricated bands, 3D-printed appliances provide a more precise fit to the anatomical contours of the abutment teeth, reducing the risk of gingivitis and debonding caused by poor band adaptation or cement dissolution, and improving the retention and longevity of space maintainers [38,39]. In the present case, regular follow-up visits were maintained throughout treatment to assess appliance retention, oral hygiene, soft-tissue condition, and preservation of the eruption space. The patient used two consecutive space maintainers for more than 20 months, during which no debonding, soft-tissue irritation, or hygiene-related complications were observed.

The present case achieved spontaneous eruption of the successor permanent tooth and a favorable occlusion. By interceptive management implemented at the appropriate developmental stage, this approach reduced the likelihood of future invasive interventions such as orthodontic traction, or prosthetic or implant-based rehabilitation. Collectively, these outcomes highlight the importance of early diagnosis and timely intervention, with attention to the treatment timing.

There are several limitations of this case report. First, the absence of CBCT imaging and histopathological analysis limited the characterization of the PEIR lesion. CBCT was not performed because conventional radiographs were considered sufficient for clinical decision-making and additional radiation exposure was avoided in this pediatric patient. Furthermore, although the clinical and two-dimensional radiographic presentations were highly indicative of the condition, the potential for observer bias in two-dimensional radiographic interpretation remains. It cannot be confirmed that the lesion of tooth 75 was definitively restricted in the crown, so it was described as suspected PEIR throughout this report. Future studies with larger sample sizes and rigorous histological evaluations are warranted to further elucidate the concurrent pathogenesis and diagnostic consensus of severe ankylosis and PEIR.

## 5. Conclusions

The management of ankylosed primary teeth relies on individualized strategies, based on the severity of infraocclusion, progression of associated lesions, and the successor’s developmental stage. Although PEIR in primary teeth is rare, undetected lesions may later present as cavitated caries or pulpal infection. Therefore, careful radiographic monitoring remains important in suspicious cases. In this case, early interceptive orthodontic treatment and timely extraction of the ankylosed molar successfully corrected the ectopic eruption, restored arch space, and facilitated the spontaneous eruption of the permanent successor. Early diagnosis, timely management, and tailored interventions are crucial to prevent severe malocclusion and minimize the need for complex interventions for the later stages.

## Figures and Tables

**Table 1:** Cephalometric data of the West China method.

Measurement	Pretreatment	Posttreatment	Mean Normal	Standard Deviation
SNA	80.55	79.81	82	4
SNB	75.35	75.29	78	4
ANB	5.2	4.52	4	2
FMIA (L1-FH)	57.8	58.09	53	6
FMA (FH-MP)	22.16	22.1	30	4
IMPA (L1-MP)	100.04	99.81	93.9	6.2

## Data Availability

All original data and contributions associated with this study are contained within the manuscript.

## Abbreviations

The following abbreviations are used in this manuscript:

FMA	Frankfort–mandibular plane angle
FMIA	Frankfort–mandibular incisor angle
IMPA	Incisor–mandibular plane angle
SNA	Sella–nasion–A point angle
SNB	Sella–nasion–B point angle
ANB	A point–nasion–B point angle
PEIR	Pre-eruptive intracoronal resorption
SLS	Selective laser sintering
